# Improving Sternal Closure Outcomes in Cardiac Surgery: Polyethylene Suture Tapes vs. Steel Wires

**DOI:** 10.3390/jcm14010277

**Published:** 2025-01-06

**Authors:** Zain Khalpey, Ujjawal Aditya Kumar, Usman Aslam, Tyler Phillips, Zacharya Khalpey, Anthony Cooper, Robert Riley

**Affiliations:** 1 Department of Cardiac Surgery, HonorHealth, 10210 N 92nd St, Scottsdale, AZ 85258, USA; ujjawal.lab@khalpey.ai (U.A.K.); uaslam@honorhealth.com (U.A.); tylerphilly14@gmail.com (T.P.); antcooper@honorhealth.com (A.C.); rriley@honorhealth.com (R.R.); 2 Khalpey AI Lab, Applied and Translational AI Research Institute (ATARI), 10210 N 92nd St, Scottsdale, AZ 85258, USA; zach@khalpey.ai; 3 School of Clinical Medicine, University of Cambridge, Hills Road, Cambridge CB2 0SP, UK; 4 Department of General Surgery, HonorHealth, Phoenix, AZ 85020, USA

**Keywords:** cardiac surgery, median sternotomy, open-heart surgery, suture tapes

## Abstract

**Background:** Steel wires are often inadequate for sternal closure for patients at high risk of sternal complications. This study compares a novel sternal closure system to conventional steel wires to assess its potential to reduce sternal complication rates and improve clinical outcomes. **Methods:** A retrospective study was conducted on 300 consecutive patients undergoing cardiac surgery via median sternotomy. A total of 150 patients underwent steel wire sternal closure, while 150 underwent suture tape closure. Preoperative, intraoperative, and postoperative data were collected and analyzed for both groups. **Results:** Rates of sternal wound infections (1% vs. 5%, *p* = 0.0363) and sternal dehiscence (0% vs. 5%, *p* = 0.0297) were lower in the suture tape group. Suture tape patients had significantly less pain at 14 and 30 days (*p* = 0.0002 and 0.0071). The requirement for sternal protection adjuncts was eliminated with suture tape closure. Sternal closure time was significantly shorter in the suture tape group (11 vs. 19 min, *p* < 0.0001). **Conclusions:** Suture tapes proved safe, feasible, and effective for sternal closure, demonstrating significant advantages for sternal closure over steel wires, with reduced rates of sternal dehiscence, infection, postoperative incisional pain, and closure time. These superior outcomes and the elimination of sternal protection adjuncts can potentially reduce healthcare costs. Our experiences suggest that this novel sternal closure system has significant potential, with larger prospective studies warranted to optimize sternal closure strategies.

## 1. Introduction

Advances in surgical techniques and anesthesia have allowed higher-risk patients to undergo cardiac surgery via median sternotomy. For high-risk patients, however, conventional sternal closure with steel wires may be inadequate, resulting in complications like sternal wound infection or dehiscence. Sternal wound dehiscence typically occurs within the first two postoperative weeks [1], affecting up to 8% of median sternotomies with a mortality rate of up to 9% [2]. Given that such sternal complications are a severe complication of open cardiac surgery, optimizing sternal closure is crucial.

Identifying high-risk patients for sternal complications is essential for improving postoperative outcomes. Risk factors include obesity, diabetes mellitus, chronic obstructive pulmonary disease (COPD), bilateral or pedicled internal mammary artery use, prolonged cardiopulmonary bypass time, and reoperation for bleeding [3,4]. Obese patients are particularly vulnerable, with risk increasing as BMI rises [5]. Excess weight puts greater mechanical stress on the sternum, increasing the likelihood of wire loosening during basic activities like breathing and coughing. Increased mechanical stress can compromise sternal reapproximation and cause bone cut-through, leading to sternal weakness, instability, and poor wound healing. COPD patients, prone to frequent and forceful coughing, are also at high risk. Patients with a history of smoking or diabetes often have microvascular changes impairing sternal perfusion and wound healing.

Addressing the mechanical component is particularly important in overweight patients [6], as sternal wound dehiscence usually occurs before bone healing. Elderly, frail, or osteoporotic patients are also at increased risk. Frail patients have a two-fold higher risk of sternal complications compared to non-frail patients [7]. Osteoporosis or long-term steroid therapy can compromise bone quality, further increasing the risk of sternal instability. Recognizing these high-risk populations allows surgeons to implement targeted strategies to mitigate sternal complications and improve overall outcomes.

We present a novel system offering a superior approach to conventional steel wire closure in high-risk cardiac surgery patients. FiberTape and TigerTape (Arthrex, Inc., Naples, FL, USA) are innovative suture tapes with an ultrahigh-molecular-weight polyethylene core coated with a braided polyester and silicone composite. While they are widely used in orthopedic surgery (e.g., rotator cuff repair, bone cerclage for fracture repair [8]), their use in cardiac surgery is currently limited. These suture tapes offer potential advantages over conventional steel sternal wires. Their flat, rectangular profile provides a broader contact area with the sternum than sternal wires, potentially reducing the risk of tissue cut-through (“cheese-wiring”). They, therefore, provide strength to the sternal cerclage while preserving bone integrity.

Biomechanical testing has demonstrated the suture tapes’ superior strength compared to stainless-steel wires. In figure-of-eight load-to-failure tests, suture tapes withstood 3448 Newtons, nearly double the 1760 Newtons steel wires withstood. Similar results were observed in simple loop configuration tests (suture tapes: 1750N, steel wires: 815N) [9]. These findings suggest the novel system is approximately twice as strong as conventional steel sternal wires. Further biomechanical analysis confirmed the suture tape system’s ability to withstand greater forces, demonstrating its superiority in maintaining sternal stability [10]. Additionally, steel implants, especially cerclages, have the potential for increased corrosion due to friction, possibly leading to connective tissue encapsulation and increased risk of postoperative infections and bone instability [11].

With the increasing risk of cardiac surgical patients and the resulting need for improved sternal closure systems, this study aims to evaluate the feasibility of suture tape sternal closure and its potential to improve patient outcomes and reduce complications after median sternotomy.

## 2. Patients and Methods

This is a retrospective cohort study comprising 300 consecutive patients who underwent cardiac surgery via median sternotomy by a single surgeon at our institution between July 2022 and January 2024. As part of an enhanced wound healing protocol, the surgeon in question moved to using the suture tapes as part of routine practice in April 2023. The wire cohort in this study are the last 150 patients that underwent surgery via sternotomy before this practice change (July 2022 to April 2023), and the suture tape cohort in this study are the first 150 patients to undergo sternal closure using the new system (April 2023 to January 2024). Any patients with previous sternal complications or those under the age of 18 were excluded. Institutional Review Board ethical approval was granted for outcome analysis in this study (IRB#23-0025, granted 26 April 2023), and informed consent was obtained from all patients for the relevant surgical procedures and anonymized study inclusion. All methods of this study were conducted per the applicable guidelines and regulations for working with human subjects.

### 2.1. Data Collection

Demographic data, preoperative clinical characteristics, operative characteristics, and postoperative outcomes were collected from the institutional electronic health record system (Epic) [12]. Data collected were anonymized and stored on a secure server per institutional information governance protocols for outcomes research data. Preoperative characteristics collected also included comorbidities that presented a risk for postoperative sternal complications such as a high BMI (obesity), diabetes mellitus (DM), chronic obstructive pulmonary disease (COPD), a smoking history, prior sternotomy, and any long-term immunosuppressive medications such as steroids. The usual operative times (cardiopulmonary bypass and aortic cross-clamp) and the total closure time were recorded. Postoperatively, data were collected for hospital mortality, sternal wound infection, and sternal wound dehiscence, defined using STS criteria [13]. Data were collected for institutional costs of sternal wound closure systems and any additional sternal protection adjuncts used, such as support vests or negative pressure dressings.

Following our institutional protocol, patients underwent follow-up evaluations at 14 and 30 days post-surgery by the cardiac surgical team. These visits included a comprehensive multi-system assessment and medication review. Significant pain was recorded if the patient reported substantial incisional pain limiting daily activities and postoperative recovery or if a new opioid medication, not used preoperatively, had been prescribed. A physical examination of the wound and a pain evaluation were conducted during follow-up appointments. The surgeon applied pressure to the manubrium and all interspaces to check for pain elicitation. Additionally, with the patient standing, lateral rotation of the thoracic spine was performed while the surgeon placed two fingers on the manubrium to assess for pain.

### 2.2. Operative Technique

**Figure 1:**
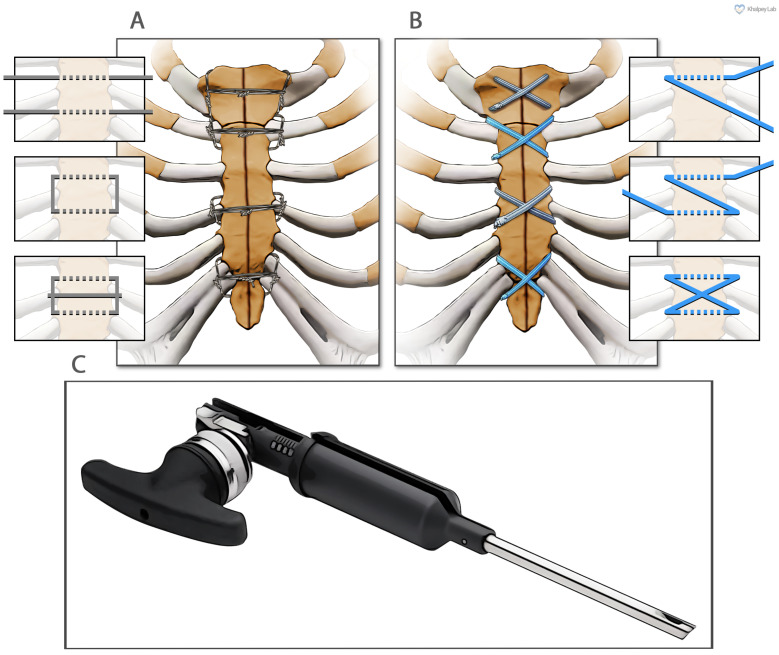
**The sternal closure approaches utilized in this study.** (**A**) Semi-Robicsek figure-of-eight sternal wire cerclage. (**B**) Suture tape sternal cerclage. (**C**) Suture Tape Tensioner Device (Arthrex Inc., Naples, FL, USA).

The treatment for the two groups diverged after the surgical procedure was completed, and the attention was turned to sternal reapproximation. The sternum was closed using surgical steel wires in a semi-Robicsek figure-of-eight pattern in the wire group (Figure 1A). Eight individual sternal wires were passed under the sternum from one side to the other in the sternal interspaces and on either side of the manubrium. On each side, adjacent pairs of wire ends were twisted together over the respective costal cartilage. Finally, each twisted wire pair on one side was tied to its corresponding twisted pair on the opposite side, creating a figure-of-eight configuration across the sternum. For the suture tape group, ultrahigh-molecular-weight polyethylene suture tapes were used, which were pre-soaked in vancomycin solution for five minutes before use. The TigerTape, black and white, and FiberTape, blue and white, measured 2 mm in width. In figure-of-eight patterns, four sutures were placed around the manubrium and through the sternal interspaces around the sternum (Figure 1B). A tensioner (Figure 1C) was then used to tighten each figure-of-eight complex sequentially, approximating the sternum with 60 to 80 lb of pressure. Finally, a half-hitch knot was tied to lock/secure the sutures.

### 2.3. Statistical Analysis

Data were summarized using descriptive statistics. For continuous variables, parametric data were presented as the mean and standard error in the mean (SEM), while non-parametric data used median and interquartile range (IQR). Categorical variables were expressed as N (%). Group comparisons used *t*-tests for parametric and Mann–Whitney tests for non-parametric continuous variables. Categorical variables were compared using Chi-Square or Fisher exact tests. All statistical analyses were performed using R v4.4.1 (R Foundation, Vienna, Austria) [14]. *p*-values were calculated by comparing the suture tape and wire groups with a conventional significance threshold of 0.05.

## 3. Results

### 3.1. Comparison of Preoperative Characteristics

Group comparisons of patient characteristics are shown in Table 1.

Comparison of preoperative characteristics revealed no significant demographic differences between groups. Comorbidity rates were similar between groups. As expected, obesity was the most prevalent comorbidity in both groups, followed by either smoking history or diabetes mellitus. The similar comorbidity profiles between groups suggest comparable sternal risk profiles, implying that observed differences in outcomes were likely due to differing sternal closure approaches between groups.

### 3.2. Comparison of Operative Characteristics

Operative characteristics (case mix, cardiopulmonary bypass, and aortic cross-clamp times) were generally similar between wire and suture tape closure groups (Table 2).

**Figure 2:**
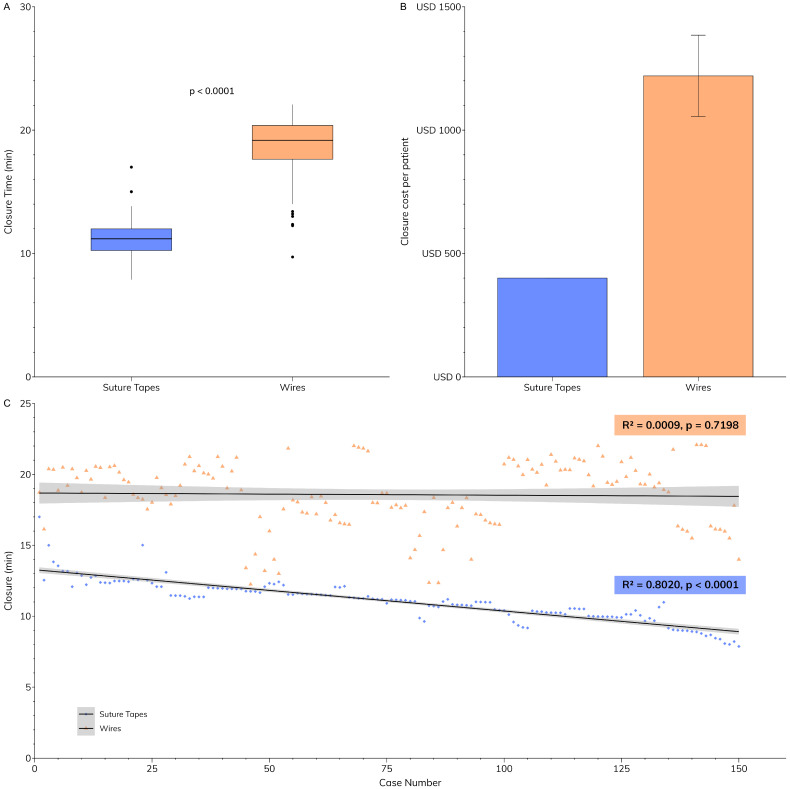
**Comparison of closure characteristics.** (**A**) Closure was significantly faster in the suture tape group compared to the wire closure group. (**B**) Comparison of closure costs per obese patient. (**C**) Consecutive closure times for suture tape and sternal wire groups. Closure times in the suture tape group decreased over 150 cases, with minimal variation from the trendline, whereas there was significant variation in the wire closure group.

All patients undergoing coronary artery bypass involving the left internal mammary artery (LIMA) underwent skeletonized harvesting to better preserve sternal perfusion and optimize postoperative healing [3]. Closure time was significantly shorter with suture tape closure than with wire closure (Figure 2A, 11 vs. 19 min, *p* < 0.0001). Within the suture tape group, closure time significantly decreased over the first 150 cases (R^2^ = 0.8020, *p* < 0.0001), with very little variation from the trend as shown in Figure 2C. However, the wire closure group showed no significant trend (R^2^ = 0.0009, *p* = 0.7198), though significant variation in closure time was seen.

### 3.3. Comparison of Postoperative Outcomes

When postoperative outcomes are compared between groups (Table 3), the suture tape closure group had far superior outcomes compared to the wire closure group.

The suture tape closure group had significantly lower rates of sternal wound infection (1% vs. 5%, *p* = 0.0363) and dehiscence (0% vs. 5%, *p* = 0.0297). There were also significantly fewer patients experiencing significant incisional pain at the 14-day (0% vs. 9%, *p* = 0.0002) and 30-day (0% vs. 5%, *p* = 0.0071) follow-ups, respectively. Additionally, hospital mortality was significantly lower in the suture tape closure group (1% vs. 7%, *p* = 0.0349). In addition to these superior outcomes, the suture tape closure group did not require additional adjuncts to stabilize and protect the sternal wound. These included technologies such as negative pressure dressings and specialized chest binders/support vests used in all patients in the wire closure group. While these were used in the first few patients in the suture tape closure group, these were quickly phased out as there was deemed to be no need for their use, given the advantages of the suture tape closure system.

### 3.4. Financial Comparison

Our study found that sternal closure costs varied significantly between methods. For wire closure with necessary adjuncts in obese patients, costs ranged from USD 1055 to USD 1385 per patient. This cost includes USD 80 to USD 160 for sternal wires (eight per patient, USD 10 to USD 20 each [15]), USD 225 for a support vest [16], and USD 750 to USD 1000 for 3 days of Prevena system use (therapy unit and dressing costing between USD 500 and USD 600, the dressing kit costing between USD 200 and USD 300, and the canister for fluid collection costing between USD 50 and USD 100) [17]. In contrast, suture tape closure costs USD 400 per obese patient (four tapes, USD 100 each [18]). This practice change resulted in savings of USD 655 to USD 985 per obese patient compared to wire closure. Figure 2B compares the per-patient closure costs, clearly showing the cost advantage of suture tapes over wires for obese patients.

For the 88 obese patients in the wire group, total costs were USD 92,840 to USD 121,880. Had similar adjuncts been needed for the 84 obese patients in the suture tape group, costs would have been USD 115,500 to USD 136,500. However, these were not deemed necessary due to the suture tape system’s stability, reducing actual expenditure to just USD 33,600, a saving of up to USD 102,900. This analysis demonstrates significant cost savings with the suture tape system, primarily by eliminating the need for additional support devices in obese patients.

## 4. Discussion

Our study demonstrates the efficacy and safety of the suture tape sternal closure system in cardiac surgery patients. This novel approach significantly reduced sternal complications, operative time, and postoperative pain compared to conventional steel wire closure. The patient cohort in this study generally represented a high-risk population for sternal complications, including many individuals with obesity, frailty, diabetes, and other comorbidities known to compromise wound healing and sternal stability [19]. Despite these risk factors, our results show remarkably low rates of sternal wound infections (1% vs. 5%, *p* = 0.0363) and sternal dehiscence (0% vs. 5%, *p* = 0.0297) in the suture tape group compared to the wire closure group. These findings are particularly significant given the vulnerability of our patient population.

One of the most striking outcomes was the complete absence of significant postoperative pain at 14-day and 30-day follow-ups in the suture tape group, compared to 9% and 5% in the wire group. This reduction in pain likely contributes to improved patient comfort, earlier mobilization, and potentially faster recovery. The mechanism behind this pain reduction may be attributed to the broader, flatter profile of the suture tapes, which could reduce localized pressure and tissue damage compared to traditional wires. Additionally, eliminating chest stabilization adjuncts such as the specialized binders/support vests aided in pulmonary rehabilitation, as these adjuncts were often found to restrict chest expansion. On balance, they were previously thought to be advantageous due to the benefits of protecting the sternum postoperatively, but the ability to eliminate them from our practice was a significant advantage of switching to a suture tape closure system.

The significantly lower hospital mortality rate in the suture tape group (1% vs. 7%, *p* = 0.0349) is a striking finding that warrants further investigation. While this outcome may be multifactorial, it suggests that the benefits of the suture tape system could extend beyond wound healing and pain reduction to impact overall patient survival. Operatively, the suture tape system significantly decreased closure time (11 vs. 19 min, *p* < 0.0001). This efficiency reduces operative time (and cost) and potentially decreases anesthesia exposure and associated risks. Unlike the wire closure group, which exhibited considerable variability, the suture tape group showed minimal variation in closure times. Unlike steel sternal wires, suture tapes delivered reliable, reproducible, and consistent sternal closure, whereas wires were less predictable and often required adjustments to achieve suitable sternal closure.

Our findings align with and expand upon previous research in this area. Coster and colleagues reported similar success using this system in bilateral lung transplant via transverse thoracosternotomy, another high-risk population for sternal complications [20]. The reproducibility of positive outcomes across different surgical contexts strengthens the case for broader adoption of this sternal closure system.

### 4.1. Advantages of Suture Tape Sternal Closure

The suture tape system offers several advantages over steel wire closure, with significant biomechanical advantages. Previous studies have shown that these suture tapes can withstand nearly double the force of steel wires before failure [9,21]. The greater strength and flat, broader contact area compared to steel wires likely contribute to our study’s reduced incidence of sternal dehiscence. The system’s ability to maintain appropriate tension while lowering the risk of tissue cut-through addresses a critical weakness of traditional wire closure, particularly in patients with poor bone quality or high mechanical stress on the sternum, such as obese patients. Furthermore, the tensioner device allows for controlled, calibrated, and reproducible tensioning of the suture tapes, optimizing sternal reapproximation and closure.

Compared to sternal wire tips (even with turnback), the flatter knot stack reduces patient discomfort and is less noticeable on the chest wall, which likely contributed to the significant difference in postoperative pain between groups in this study. In emergent situations requiring chest reopening, such as cardiac arrest, the suture tapes can be quickly cut using standard surgical tools like a scalpel or scissors, eliminating the need for specialized wire-cutters and resternotomy saws, which add the additional risk of damaging the underlying structures. This ease of reopening saves time and increases safety during emergency resternotomies, which are already time-critical and high-risk events. Additionally, the suture tapes’ lower profile and flat-to-bone design allow sternal reinforcement with fixation plate systems if required, as these can be easily implanted above and around the suture tapes, which cannot be done with steel wires.

Additionally, the suture tape system mitigates the risk of wire-stick injuries to surgical staff, which, although rare, represents an important safety consideration. Our practice of pre-soaking the suture tapes in vancomycin solution before use may have contributed to the low infection rates observed. This approach, combined with the material properties of the suture tapes, appears to create an environment less conducive to bacterial colonization than steel wires. Lastly, eliminating the need for sternal protection adjuncts (e.g., negative pressure dressings, specialized chest binders) in the suture tape group further simplifies postoperative care and potentially reduces associated costs.

### 4.2. Financial Considerations

The suture tape system’s cost advantages may offset its higher initial price than steel wires. While more expensive upfront, the suture tape system’s overall cost savings justify its use, especially for obese patients. It eliminates the need for sternal protection adjuncts required with wire closure, such as Posthorax chest support vests (SternumBrace Inc., Clearwater, FL, USA) and Prevena negative pressure dressings (3M Medical, St. Paul, MN, USA), which were necessary for all obese patients in the wire group.

Shorter operating room times with the suture tape system reduce anesthesia exposure and improve surgical resource efficiency. Lower complication rates may also enhance quality metrics (e.g., STS) and potentially increase hospital reimbursement. The reduced complications and postoperative pain observed in the suture tape group could lead to shorter hospital stays, further decreasing healthcare costs. Although a formal cost-effectiveness analysis is ongoing, our preliminary financial analysis suggests the suture tape system offers significant economic benefits alongside its clinical advantages.

### 4.3. Limitations

Despite these promising results, our study has some limitations. As a single-center, retrospective analysis, it may have inherent biases and limited generalizability, though the well-matched study groups hopefully mitigate this concern. Longer-term follow-up is needed to assess outcome durability. A formal cost-effectiveness analysis comparing suture tape to wire closure, including potential savings from reduced complications and shorter hospital stays, would inform healthcare systems considering this technology. While we assessed significant pain at follow-up through various methods, these were not quantitative. To address this, we are developing a comprehensive Cardiac Surgery Pain Assessment Scale, incorporating numerical rating scales for sternal pain, sternum-related patient anxiety, and sternal instability.

In conclusion, our study shows the suture tape sternal closure system is a safe, effective alternative to conventional steel wires for high-risk cardiac surgery patients. Its biomechanical advantages, reduced operative time, and improved postoperative outcomes make it a promising innovation. While prospective, multicenter studies are needed to establish long-term efficacy and cost-effectiveness, our data provide compelling evidence for its potential to improve cardiac surgery outcomes, especially in high-risk patients. As we refine surgical techniques to meet the needs of an increasingly complex patient population, innovations like this represent considerable development in optimizing patient care and outcomes.

## 5. Conclusions

The suture tape sternal closure system demonstrates significant advantages over conventional steel wire closure in cardiac surgery patients, particularly those at high risk for sternal complications. This novel approach resulted in significantly lower rates of sternal wound infections (1% vs. 5%, *p* = 0.0363), sternal dehiscence (0% vs. 5%, *p* = 0.0297), and hospital mortality (1% vs. 7%, *p* = 0.0349). The system also eliminated postoperative pain at both 14- and 30-day follow-ups and significantly reduced closure time (11 vs. 19 min, *p* < 0.0001). Furthermore, the elimination of sternal protection adjuncts in the suture tape group led to substantial cost savings, particularly in obese patients. While larger prospective studies with longer follow-up periods are needed, these results suggest that the suture tape closure system represents a significant advancement in sternal closure technique, offering improved patient outcomes and potential healthcare cost reductions. The system’s superior biomechanical properties, combined with its ease of use and consistent performance, make it a promising innovation for optimizing cardiac surgical care, especially in high-risk populations.

## Figures and Tables

**Table 1:** **Comparison of preoperative patient characteristics**. Both groups were similar.

Variable	Total	Wire Closure	Suture Tape Closure	*p*-Value
Number	300	150	150	N/A
Age (years)	67 ± 11	67 ± 11	66 ± 11	0.2333
Sex: Male	211 (70%)	109 (73%)	102 (68%)	0.8261
STS Score	1.625 (0.940–2.728)	1.625 (0.915–2.797)	1.480 (0.984–2.047)	0.9633
STS DSWI Score	0.160 (0.100–0.289)	0.160 (0.100–0.290)	0.182 (0.120–0.265)	0.9038
BMI (kg/m^2^)	32.70 ± 8.79	33.31 ± 8.96	32.09 ± 8.61	0.2302
Obese	172 (57%)	88 (59%)	84 (56%)	0.7263
DM	88 (29%)	36 (24%)	52 (35%)	0.0568
COPD	14 (5%)	6 (4%)	8 (5%)	0.7855
Smoking History	90 (30%)	43 (29%)	47 (31%)	0.7056
Prior Sternotomy	6 (2%)	4 (3%)	2 (1%)	0.6843
Immunosuppressive Medications	26 (9%)	11 (7%)	15 (10%)	0.5389

**Table 2:** **Comparison of operative characteristics**. Similar case mix, cardiopulmonary and aortic cross-clamp times. Significantly shorter closure time in suture tape group.

Variable	Total (*n* = 150)	Wire Closure (*n* = 150)	Suture Tape Closure (*n* = 150)	*p*-Value
Procedure Type				0.3424
CABG	154 (51%)	80 (53%)	74 (49%)	
Valve ± Maze	85 (28%)	37 (25%)	48 (32%)	
CABG and Valve ± Maze	29 (10%)	16 (11%)	13 (9%)	
Aortic	30 (10%)	17 (11%)	13 (9%)	
Other	2 (1%)	0 (0%)	2 (1%)	
Cardiopulmonary Bypass (min)	95 ± 48	101 ± 54	90 ± 39	0.0787
Aortic Cross-Clamp (min)	67 ± 30	66 ± 33	68 ± 27	0.4861
Closure (min)	13 (11–19)	19 (18–20)	11 (10–12)	<0.0001
LIMA Skeletonization	162 (100%)	83 (100%)	79 (100%)	>0.9999

**Table 3:** **Comparison of postoperative outcomes between study groups**. The suture tape group had significantly better outcomes—significantly lower hospital mortality, sternal wound infection, and dehiscence. Fewer patients with significant pain at 14- and 30-day follow-up. Length of ICU and hospital admissions were similar between groups.

Outcome	Total (*n* = 300)	Wire Closure (*n* = 150)	Suture Tape Closure (*n* = 150)	*p*-Value
ICU LOS	3.0 (2.0–5.0)	3.0 (2.0–5.0)	2.5 (2.0–4.5)	0.7455
Hospital LOS	7.00 (6.00–11.0)	8.0 (6.0–11.0)	7.0 (5.0–11.0)	0.2656
Hospital Death	12 (4%)	10 (7%)	2 (1%)	0.0349
Sternal Wound Infection	9 (3%)	8 (5%)	1 (1%)	0.0363
Sternal Wound Dehiscence	7 (2%)	7 (5%)	0 (0%)	0.0297
Significant Pain @ 14d	13 (4%)	13 (9%)	0 (0%)	0.0002
Significant Pain @ 30d	8 (3%)	8 (5%)	0 (0%)	0.0071

## Data Availability

All data can be made available upon reasonable request to Dr. Zain Khalpey (zain@khalpey.ai).
